# Primary Prevention of Cardiovascular Disease and Type 2 Diabetes Mellitus Using Mobile Health Technology: Systematic Review of the Literature

**DOI:** 10.2196/21159

**Published:** 2020-10-29

**Authors:** Vera Helen Buss, Stuart Leesong, Margo Barr, Marlien Varnfield, Mark Harris

**Affiliations:** 1 Centre for Primary Health Care and Equity University of New South Wales Sydney Australia; 2 Australian e-Health Research Centre CSIRO Brisbane Australia

**Keywords:** systematic review, mobile health, telemedicine, primary prevention, cardiovascular diseases, diabetes mellitus, type 2

## Abstract

**Background:**

Digital technology is an opportunity for public health interventions to reach a large part of the population.

**Objective:**

This systematic literature review aimed to assess the effectiveness of mobile health–based interventions in reducing the risk of cardiovascular disease and type 2 diabetes mellitus.

**Methods:**

We conducted the systematic search in 7 electronic databases using a predefined search strategy. We included articles published between inception of the databases and March 2019 if they reported on the effectiveness of an intervention for prevention of cardiovascular disease or type 2 diabetes via mobile technology. One researcher performed the search, study selection, data extraction, and methodological quality assessment. The steps were validated by the other members of the research team

**Results:**

The search yielded 941 articles for cardiovascular disease, of which 3 met the inclusion criteria, and 732 for type 2 diabetes, of which 6 met the inclusion criteria. The methodological quality of the studies was low, with the main issue being nonblinding of participants. Of the selected studies, 4 used SMS text messaging, 1 used WhatsApp, and the remaining ones used specific smartphone apps. Weight loss and reduction in BMI were the most reported successful outcomes (reported in 4 studies).

**Conclusions:**

Evidence on the effectiveness of mobile health-based interventions in reducing the risk for cardiovascular disease and type 2 diabetes is low due to the quality of the studies and the small effects that were measured. This highlights the need for further high-quality research to investigate the potential of mobile health interventions.

**Trial Registration:**

International Prospective Register of Systematic Reviews (PROSPERO) CRD42019135405; https://www.crd.york.ac.uk/PROSPERO/display_record.php?RecordID=135405

## Introduction

### Description of the Condition

Worldwide, chronic diseases are the main cause of death and years lived with disability [1,2]. Cardiovascular disease (CVD) and type 2 diabetes mellitus (T2DM) are globally among the top 5 chronic conditions in terms of incidence and prevalence [2]. The behavioral risk factors for these conditions, such as smoking, harmful use of alcohol, poor diet, and physical inactivity, are highly correlated with the disease progression [3]. For example, Gellert et al [4] observed in their meta-analysis a dose-response relationship between the number of cigarettes smoked and premature death. They also found an inverse correlation between time since cessation and all-cause mortality. Wood et al [5] reported that all-cause mortality was positively associated with the level of alcohol intake, based on data from over half a million current drinkers. Chudasama et al [6] found a negative dose-response relationship between physical activity levels and all-cause mortality in their analysis of almost half a million people. Regarding low whole-grain intake, which is the highest risk factor related to poor diet, in their meta-analysis, Zhang et al [7] showed an inverse dose-response relationship between whole-grain intake and all-cause mortality. Hence, targeting these with preventive measures could significantly reduce people’s chronic disease risk [8], and behavior change interventions are well suited for preventing CVD and T2DM [2,3].

### Description of the Intervention

To stop noncommunicable diseases from rising further, the World Health Organization (WHO) developed the Global Action Plan 2013-2020 [8]. In this report, the WHO emphasized the importance of early screening and the implementation of preventive programs. Further, the WHO recommended the use of information and communication technologies, such as the internet and mobile phone technologies, to deliver health education and promotion programs. In 2019, the WHO released a guideline with recommendations on digital interventions for health system strengthening [9]. This report outlined how the implementation of technology could overcome current challenges in health care systems and help to achieve the goal of universal health coverage. Health apps have promising potential. Wilson [10] pointed out that digital health interventions have the advantage of being easily accessible and cost-effective. According to the Pew Research Center [11], many people use their smartphones daily. Riley et al [12] reported that new advancements allow apps to be tailored to personal needs and preferences, as well as the integration of dynamic feedback systems. Despite the promising potential of health apps, there is still ambiguity about their effectiveness, as outlined by the WHO guideline [9].

### Objective

The aim of this systematic literature review was to assess the current evidence regarding the effectiveness of mobile health–based interventions in reducing the risk for CVD and T2DM. The focus was on multiple behavioral risk–factor interventions, rather than single risk–factor interventions, because of the lack of evidence on their combined effectiveness compared with substantial evidence on single risk–factor interventions [13,14].

## Methods

### Review Standards

We conducted this systematic review in accordance with the Preferred Reporting Items for Systematic Reviews and Meta-Analyses (PRISMA) statement [15] and registered it with International Prospective Register of Systematic Reviews (PROSPERO; registration number CRD42019135405).

### Search Strategy

We searched the following medical and bioengineering databases to retrieve all relevant articles regarding preventive mobile health intervention for CVD and T2DM: EMBASE (via Ovid), Scopus, ScienceDirect, CINAHL (via EBSCOhost), MEDLINE (via Ovid), ProQuest science and technology databases, and Ei Compendex and Inspec (both via Engineering Village 2). The search strategy (Multimedia Appendix 1) included terms relating to the 2 conditions under study and the intervention; we combined the terms using Boolean operators [16] and adapted the terms to the database-specific requirements. The search included articles published from the inception of the databases until March 25, 2019. We limited the search to English- and German-language publications because these languages were proficiently spoken by the review team. We excluded review articles, conference abstracts, comments, editorials, letters to the editor, and theses. Additionally, we identified studies using “snowballing” techniques by reviewing the reference lists of articles included in the initial search and searching for other publications by authors included in the initial search [17].

### Study Selection

#### Inclusion Criteria

The study selection followed predefined inclusion criteria according to the PICOS system (Table 1). After removing duplicate publications, we reviewed all retrieved articles for eligibility, first by examining the titles and abstracts, and then the full articles if we considered the articles to be relevant in the first step. We included in the review full articles that met the inclusion and exclusion criteria. The steps described above were performed by 1 researcher (VHB). For the title and abstract screening, a 10% random sample of all retrieved articles was validated by a second researcher (shared between the remaining researchers). If discrepancies occurred, a third researcher resolved the issue. A second researcher (SL) independently assessed which of the full articles fulfilled the inclusion and exclusion criteria. The results were compared, and discrepancies were resolved by involving a third researcher (MB).

**Table 1:** Inclusion criteria according to the PICOS system.

Criteria	Description of inclusion criteria
Participants	Adults who are free of CVD^a^ or T2DM^b^.
Intervention	Health promotion interventions that use mobile health technology (ie, mobile app or SMS text messaging) aiming to change more than 1 risk factor for 1 of the 2 chronic conditions under study.
Comparator	No intervention (ie, standard care), or waitlist control, or intervention delivered in person.
Outcome	Onset of disease (CVD or T2DM) or relative risk reduction, which can be in the form of surrogate parameters.
Study design	Randomized controlled trial, case-control study, or interrupted time series.

^a^CVD: cardiovascular disease.

^b^T2DM: type 2 diabetes mellitus.

#### Types of Participants

Participants could either be healthy or have an increased disease risk. We excluded interventions targeting adults who were already diagnosed with CVD or T2DM (depending on the aim of the intervention, eg, for CVD prevention, people diagnosed with CVD) at baseline. Further, we excluded studies intended for minors (<18 years of age). The conditions under study were CVD and T2DM, for which we applied the following WHO definitions: CVD is a “group of disorders of heart and blood vessels,” including coronary heart disease, cerebrovascular disease, peripheral vascular disease, heart failure, rheumatic heart disease, congenital heart disease, and cardiomyopathies [18]; T2DM “is a chronic disease that occurs...when the body cannot effectively use the insulin it produces” [19].

#### Types of Intervention

We included primary studies if they evaluated the effectiveness of a mobile phone–based intervention for primary prevention of 1 of the conditions under study. The intervention had to be delivered, at least partially, via mobile health technology (ie, mobile app or SMS text messaging) with the aim of changing more than 1 risk factor for 1 or more of the chronic conditions under study. We defined a mobile app as a software program that can run on mobile devices such as smartphones, and a text message as a written message sent to a mobile phone. The type of interventions that we included needed to be aimed at health promotion using behavior change strategies, including counselling or education regarding disease-related knowledge, healthy diet, physical activity, smoking cessation, motivational messages, and goal setting. We excluded from the review studies that exclusively targeted 1 behavioral risk factor (eg, smoking only, diet only, or step count only).

#### Types of Comparator

The comparison group could consist of either no intervention (ie, standard care), or a waitlist control, or an intervention delivered in person. Studies were eligible if they included adults who were free of CVD or T2DM at study baseline, depending on the condition targeted in the study.

#### Types of Outcome

Studies were only eligible for inclusion if their main outcomes were disease incidence (either CVD or T2DM) or a reduction in disease risk, which could be measured using a risk prediction tool (such as the Framingham score for CVD [20]) or surrogate parameters. Examples of surrogate parameters were weight, waist circumference, blood pressure, blood glucose, level of physical activity, dietary intake, or smoking status. Additional outcomes that we included in the review were the feasibility of mobile health interventions, disease knowledge, and quality of life. Respective outcome measures included dropout rates, participants’ acceptability of and adherence to the intervention, and questionnaires assessing disease knowledge and quality of life.

#### Types of Study Design

We restricted the study design to randomized controlled trials (RCTs), case-control studies, and interrupted time series in order to have a measurement against which the effectiveness of the intervention could be compared.

### Data Extraction and Synthesis

Relevant data (study objective, study design, study population, comparator, description of the intervention, duration of the intervention or follow-up, outcomes, main results, and methodology for the assessment of the study’s quality) were extracted by 1 researcher (VHB) using a standardized form in Excel 365 (Microsoft Corporation). This was reviewed by all the other researchers. We synthesized the main results of the included studies in a narrative manner focusing on the intervention delivery and reported outcomes. A meta-analysis was not possible due to the small number of identified studies and the heterogeneity in interventions and outcomes.

### Literature Quality Assessment

One researcher (VHB) assessed the risk of bias using the following assessment tools: for RCTs, the Cochrane Collaboration’s tool for assessing risk of bias [21]; and for non-RCTs, the Risk of Bias In Non-randomized Studies - of Intervention assessment tool [22].

## Results

### Results of the Literature Search and Study Selection

**Figure 1:**
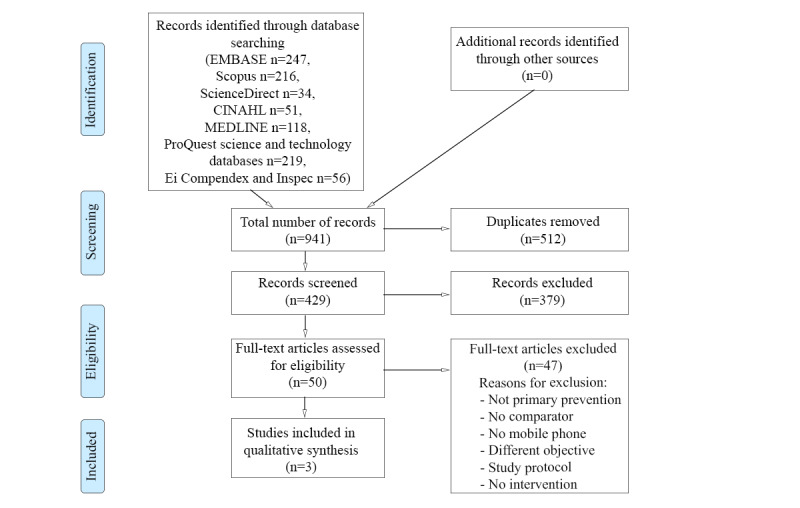
Full article selection process for cardiovascular disease.

**Figure 2:**
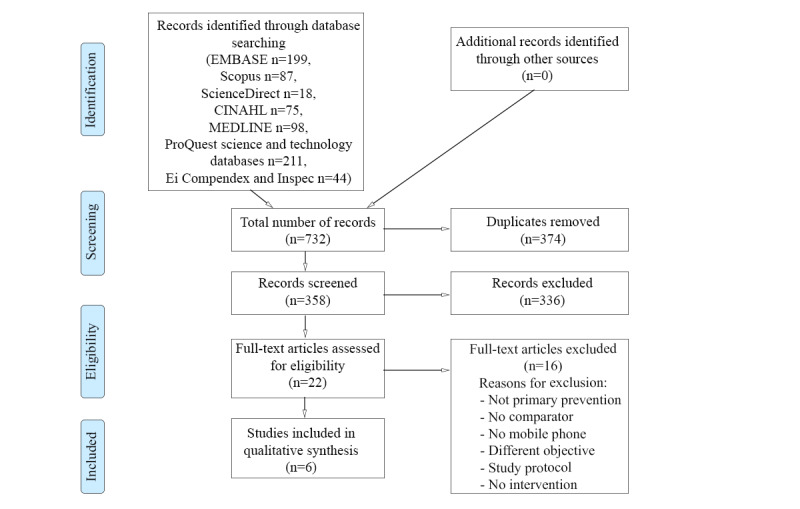
Full article selection process for type 2 diabetes.

In total, we identified 941 articles using the search strategy for CVD and 732 articles using the search strategy for T2DM. In the validation of the 10% random sample of all retrieved articles, there was a 100% agreement (after initial disagreements were resolved by a third investigator) with the selection conducted by the researcher who screened all articles. Finally, 3 CVD articles [23-25] and 6 T2DM articles [26-31] fulfilled the inclusion and exclusion criteria; we identified no additional articles through the snowballing technique (Figures 1 and 2). We excluded many articles for several of the exclusion criteria.

### Results of the Data Extraction

There were 3 CVD [23-25] and 6 T2DM studies [26-31]. Table 2 provides details about the CVD studies and Table 3 provides details about the T2DM studies. For each study, the table includes the first author, year of publication, study design and duration, objectives, study population, interventions and comparators, outcomes, and the main results.

**Table 2:** Data extraction from cardiovascular disease (CVD) studies.

First author, date, reference	Study design and duration; objectives	Study population	Intervention and comparator	Outcomes	Main results
Gore, 2019 [23]	Non-RCT^a^ for 12 months; effectiveness of an SMS text message intervention to reduce CVD risk	Adults from the United States at high risk of CVD without preexisting coronary artery disease, cerebrovascular disease, and diabetes; intervention n=204, usual care n=408	Create action plan with community health workers and return 6-12 months after initial screening for retesting; intervention: text messages once/day on advice on healthy eating, PA^b^, weight loss, contacting community health worker; control: usual care	Engagement, program retention, changes in risk factors (smoking, fat and fiber intake, PA, weight, BMI, BP^c^, low-density lipoprotein), Framingham risk score	Only statistically significant decrease in fat intake (intervention −26.3% vs control −10.6%; *P*=.001)
Muntaner-Mas, 2017 [24]	Non-RCT for 10 weeks; effectiveness of a WhatsApp-based PA intervention to reduce CVD risk factors	Spanish adults aged 53-73 years without medical conditions or other physical problems requiring special medical attention and who were able to perform rigorous PA; mobile group n=7, training group n=16, control n=9	Intervention: twice/week functional fitness for training and mobile group; for training group face-to-face sessions, for mobile group training videos for download via WhatsApp, chat function plus motivational messages from study coordinator; control: no intervention	BP, WC^d^, waist to height ratio, weight, BMI, fat mass index, fat-free mass index, heart rate after exercise, balance, handgrip strength, aerobic capacity	No statistically significant differences between mobile group and control; statistically significant differences between training group and control group (systolic BP *P*=.038; diastolic BP *P*=.005; mean arterial BP *P*=.006; heart rate after exercise *P*=.002)
Rubinstein, 2016 [25]	RCT for 12 months; effectiveness of preventive mobile health intervention in adults with prehypertension	Adults aged 30-60 years with prehypertension from poor urban settings in Argentina, Guatemala, and Peru, free of hypertension, diabetes, and CVD; intervention n=316, usual care n=321	Intervention: monthly motivational counselling calls (healthy diet and PA) followed by weekly text messages related to behavior goals and readiness to change; control: usual care	Changes in BP, weight, BMI, WC, PA, diet	Mean differences, baseline-adjusted (95% CI): weight −0.66 kg (−1.24 to −0.07), BMI −0.30 kg/m^2^ (−0.54 to −0.06), daily intake of high-sugar and -fat servings −0.75 (−1.30 to −0.20); change in BP not significant

^a^RCT: randomized controlled trial.

^b^PA: physical activity.

^c^BP: blood pressure.

^d^WC: waist circumference.

**Table 3:** Data extraction from type 2 diabetes mellitus (T2DM) studies.

First author, date, reference	Study design and duration; objectives	Study population	Intervention and comparator	Outcomes	Main results
Arens, 2018 [26]	Non-RCT^a^ for 12 months; effectiveness of app-based weight reduction program for people with metabolic syndrome	German adults aged 30-65 years treated for metabolic syndrome in 23 medical practices; intervention n=148, usual care n=85	Health goals regarding weight and PA^b^; app for feedback; physicians with access to app data could give feedback, initiate messages, or modify goals; ≤9 free classes on diet and PA; control: usual care	5% weight reduction; change in BMI	5% weight reduction (adjusted for time in study) (95% CI): 44.8% (34.1 to 57.1) in intervention vs 11.5% (4.6 to 27.0) in control; Cox proportional hazard model for time to 5% weight reduction hazard ratio 6.2 (2.4 to 16.2; *P*<.001), baseline adjusted between groups change in weight (kg) *P*=.06 and BMI (kg/m^2^) *P*=.10
Bender, 2018 [27]	RCT for 3 months plus 3 months follow-up (no control for follow-up); effectiveness of mobile phone-based weight loss intervention to reduce T2DM risk	Filipino-American overweight or obese adults from United States at increased risk for T2DM, able to walk 20 min; intervention n=33, control n=34	5 in-person sessions, daily step count via wearable device, daily food intake and weekly weight logged in app, weekly information on weight loss, PA, and diet via private Facebook page; control: waitlist	Recruitment (goal n=50), retention, 5% weight loss, changes in weight, BMI, WC^c^, FBG^d^, HbA_1c_^e^	Weight loss ≥5%: intervention 36% vs control 6%; between-group cross-level interaction (95% CI): weight −1.1%/month (−1.7 to −0.53) and −0.85 kg/month (−1.4 to −0.35), BMI −0.93 kg/m^2^ (−1.5 to −0.40), WC −4.9 cm (−7.5 to −2.6), FBG −1.4 mg/dL (−5.9 to 3.6), HbA_1c_ −0.10% (−0.21 to 0.002)
Block, 2015 [28]	RCT for 6 months plus 6 months follow-up (no control for follow-up); effectiveness of digital health intervention for T2DM risk reduction in prediabetics	Prediabetics aged 30-69 years from United States with BMI ≥27 kg/m^2^, without diabetes medication; intervention n=163, control n=176	Tailored behavioral support for PA, diet, weight loss, stress, sleep; weekly emails with goals linked to website (tracking tools, coaching, social support, competition, health advice), app and automated phone calls; control: waitlist	Decreased HbA_1c_, FBG, weight, BMI, WC, triglyceride to HDL^f^ ratio, metabolic syndrome, Framingham diabetes risk score	Mean (95% CI) HbA_1c_ −0.26% (−0.27 to −0.24) in intervention vs control −0.18% (−0.19 to −0.16), FBG −0.41 mmol/L (−0.44 to, −0.12) in intervention vs −0.21 mmol/L (−0.15 to −0.10) in control, all outcomes significantly greater in intervention than control (*P*<.001)
Fischer, 2016 [29]	RCT for 12 months; effectiveness of text message–supported T2DM prevention program	Obese and overweight adults from United States without prediabetes, English or Spanish speaking; intervention n=82, control n=81	6 text messages per week: skills, problem solving, motivation, stress reduction, recipes, web links to additional resources, PA promotion; weekly self-reported weight; eligible for individual motivational phone health coaching; control: usual care	Change in weight; percentage of participants with ≥3% or 5% weight loss, changes in HbA_1c_ and systolic BP^g^, costs per participant	Weight (95% CI) in intervention −1.2 kg (−2.5 to 0.1) vs control −0.3 kg (−1.2 to 0.7), *P*=.05; 3% weight loss absolute difference between groups 17.0%, *P*=.02; no significant difference for 5% weight loss; HbA^1c^ in intervention −0.09% (−0.2 to 0.0) vs control 0.19% (−0.1 to 0.5), systolic BP in intervention 0.35 mmHg (−2.8 to 3.5) vs control 6.4 mmHg (3.2 to 9.5)
Fukuoka, 2015 [30]	RCT for 5 months; effectiveness of mobile app-based intervention for T2DM prevention	Overweight adults aged ≥35 years from United States at high risk of diabetes; intervention n=30; control n=31	2-week run-in period before randomizing; all daily step count via pedometer; intervention: mobile version of Diabetes Prevention Program, 6 in-person sessions, app: diaries for self-monitoring of weight, PA, and caloric intake, daily reminders and messages; control: pedometer only	% change in weight and BMI; hip circumference, BP, lipid profile, glucose levels, step count, PA, caloric and fat intake	Weight (95% CI) −6.8% (−12.2 to −1.4) in intervention vs 0.3% (−2.7 to 3.3) in control; BMI −6.6% (−12.3 to −0.9) in intervention vs 0.3% (−2.7 to 3.3) in control; both *P*<.001; also significant differences in hip circumference, BP, step count, and PA for intervention vs control; no effect on lipid profile, glucose levels, caloric or fat intake
Ramachandran, 2013 [31]	RCT for 2 years; effectiveness of SMS text messaging to reduce incidence of T2DM in men with impaired glucose tolerance	Indian men aged 35-55 years with impaired glucose tolerance; intervention n=271, control n=266	All at baseline: healthy lifestyle education and written information on diet and PA, lifestyle changes prescribed; intervention: frequent reinforcing text messages, content tailored to baseline behavior; control: usual care	Incidence of T2DM; BMI, WC, BP, lipid profile, energy intake, PA	Cumulative T2DM incidence: intervention 18%, control 27%; differences in mean change (95% CI): BMI −0.05 kg/m^2^ (−0.46 to 0.37); WC 0.04 cm (−0.56 to 0.64); systolic BP 0.04 mmHg (−0.96 to 1.03); diastolic BP −0.07 mmHg (−0.64 to 0.49); total cholesterol 0.01 mmol/L (−0.08 to 0.10); HDL 0.033 mmol/L (0.011 to 0.054); triglycerides −0.08 mmol/L (−0.17 to −0.06); energy intake –43.7 kcal (−65.5 to −22.0); PA score −1.0 (−2.0 to 0.0)

^a^RCT: randomized controlled trial.

^b^PA: physical activity.

^c^WC: waist circumference.

^d^FBG: fasting blood glucose.

^e^HbA_1c_: glycated hemoglobin.

^f^HDL: high-density lipoprotein.

^g^BP: blood pressure.

### Results of the Synthesis

#### Summary

We synthesized the results of the data extraction according to the PICOS system. Table 4 summarizes CVD and T2DM data individually and in total. For each parameter, we provide a count, as well as the list of relevant references.

**Table 4:** Synthesis of findings.

Finding		Cardiovascular disease		Type 2 diabetes		Total (n)
		No. of studies	Reference	No. of studies	Reference	
**Target population**						
	General population	1	[24]	—^a^	—	1
	At risk of the disease	2	[23,25]	6	[26-31]	8
**Location**						
	Spain	1	[24]	—	—	1
	United States	1	[23]	4	[27-30]	5
	Germany	—	—	1	[26]	1
	Latin America	1	[25]	—	—	1
	India	—	—	1	[31]	1
**Intervention delivery**						
	SMS text messaging	2	[23,25]	2	[29,31]	4
	WhatsApp	1	[24]	—	—	1
	Mobile app	—	—	4	[26-28,30]	4
**Comparator**						
	Usual care	3	[23-25]	3	[26,29,31]	6
	Waitlist	—	—	2	[27,28]	2
	Pedometer only	—	—	1	[30]	1
	Face-to-face training	1	[24]	—	—	1
**Outcomes^b^**						
	Weight loss	1	[25]	3	[27,28,30]	4
	Reduced BMI	1	[25]	3	[27,28,30]	4
	Reduced waist circumference	—	—	2	[27,28]	2
	Lower fasting blood glucose/glycated hemoglobin	N/A^c^	—	1	[28]	1
	Improved diet	2	[23,25]	1	[31]	3
	Improved physical activity	—	—	2	[30,31]	2
	Improved blood pressure	—	—	1	[30]	1
**Study design**						
	Randomized controlled trial	1	[25]	5	[27-31]	6
	Nonrandomized controlled trial	2	[23,24]	1	[26]	3

^a^—: data not available.

^b^Statistically significant compared with control group.

^c^N/A: not applicable.

#### Participants

The CVD studies were conducted in Spain, the United States, and Latin America. For the T2DM studies, 1 was conducted in Germany, 1 in India, and 4 in the United States. All studies had small to medium samples, ranging from 32 to 637 participants. For CVD, 2 of the 3 studies targeted populations at higher risk of developing CVD [23,25], whereas the study by Muntaner-Mas et al [24] included healthy people. For T2DM, all studies focused on populations at increased risk of the disease.

#### Interventions

The duration of the interventions varied from 10 weeks to 2 years. In 4 studies the participants received text messages [23,25,29,31], in 1 study the intervention was delivered via WhatsApp [24], and in the remaining 4 studies a specifically developed mobile phone app was involved [26-28,30]. Only 1 intervention was delivered fully automated [28]; all other interventions included human involvement [23-27,29-31].

#### Comparators

Of the studies, 6 used usual care as the control group. In 1 trial, the control group received pedometers only [30]; 1 study had a second comparator group, additional to usual care, which received face-to-face training sessions [24]; and 2 studies used waitlist controls [27,28], meaning that the control group received the intervention after the intervention group had completed it.

#### Outcomes

The mobile phone interventions led to statistically significant weight loss compared with the control group in 4 studies [25,27,28,30], ranging from a difference of −0.66 kg (*P*=.04) over 12 months [25] to −6.2 kg for the intervention compared with 0.3 kg for the control group (*P*<.001) over 5 months [30]. The same studies reported a statistically significant decrease in BMI compared with the control group [25,27,28,30], ranging from a difference of −0.3 kg/m^2^ (*P*=.02) over 12 months [25] to −2.2 kg/m^2^ for the intervention compared with 0.1 kg/m^2^ for the control group (*P*<.001) over 5 months [30]. A smaller waist circumference due to the intervention was measured in 2 T2DM studies [27,28], from −4.56 cm for the intervention compared with −2.22 cm (*P*<.001) for the control group over 6 months [28] to a cross-level interaction of −4.9 cm (95% CI −7.5 to −2.6) over 3 months [27]. One T2DM study reported statistically significantly lower fasting blood glucose (−0.41 mmol/L in the intervention compared with −0.12 mmol/L in the control group; *P*<.001) and glycated hemoglobin levels (−0.26% in the intervention compared with −0.18% in the control group; *P*<.001) over 6 months [28]. Statistically significantly greater changes in the lipid profile were observed in the intervention group than in the control group in 2 of the T2DM trials [28,31], from a difference in mean change of high-density lipoprotein cholesterol of 0.033 mmol/L (95% CI 0.011 to 0.054) and triglycerides of −0.080 mmol/L (95% CI −0.17 to −0.06) over 2 years [31] to a triglyceride to high-density lipoprotein ratio of –0.21 in the intervention compared with 0.21 in the control group (*P*=.04) over 6 months [28]. Improved diet patterns that were statistically superior to the control group were observed in 3 studies [23,25,31], of which 2 studies aimed at CVD prevention. Improvements in physical activity were reported in 2 T2DM studies [30,31]. Blood pressure was statistically significantly improved in the intervention groups compared with the control group in 1 T2DM study [30].

#### Study Design

A total of 6 studies were RCTs [25,27-31]; the remaining 3 were non-RCTs [23,24,26].

### Results of Literature Quality Assessment

**Figure 3:**
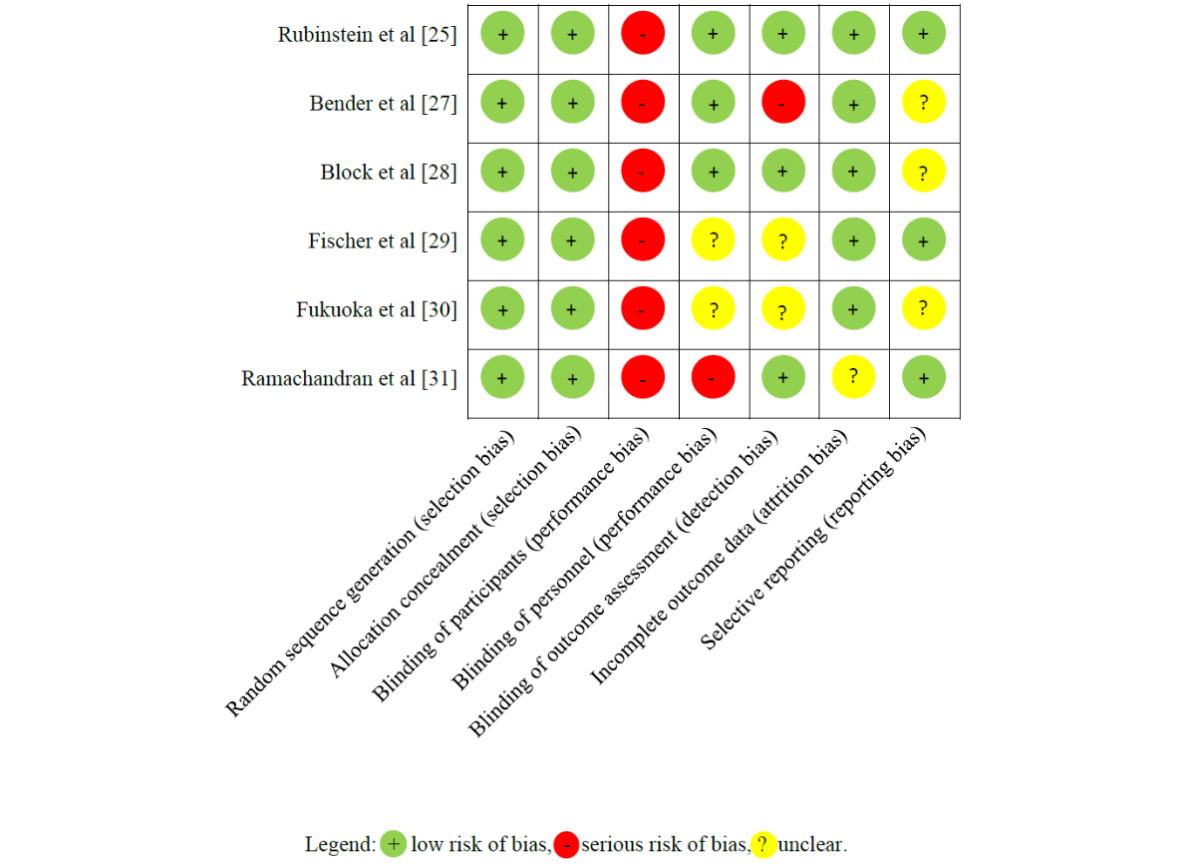
Risk-of-bias summary table for the randomized controlled trials. The upper 1 is a cardiovascular disease study and the remainder are type 2 diabetes studies.

All RCTs used acceptable methods for randomization [25,27-31], but in none of the studies were the participants blinded to the design, which is an inherent problem with this type of intervention. Figure 3 summarizes the risk of bias for the RCTs. Of the 6 studies, 3 ensured blinding of the study personnel [25,27,28] and 3 ensured the blinding of the outcome assessors [25,28,31]. Apart from the study by Fukuoka et al [30], all RCTs published study protocols on the ClinicalTrials.gov database. Overall, due to performance bias, all studies were at high risk of bias.

**Figure 4:**
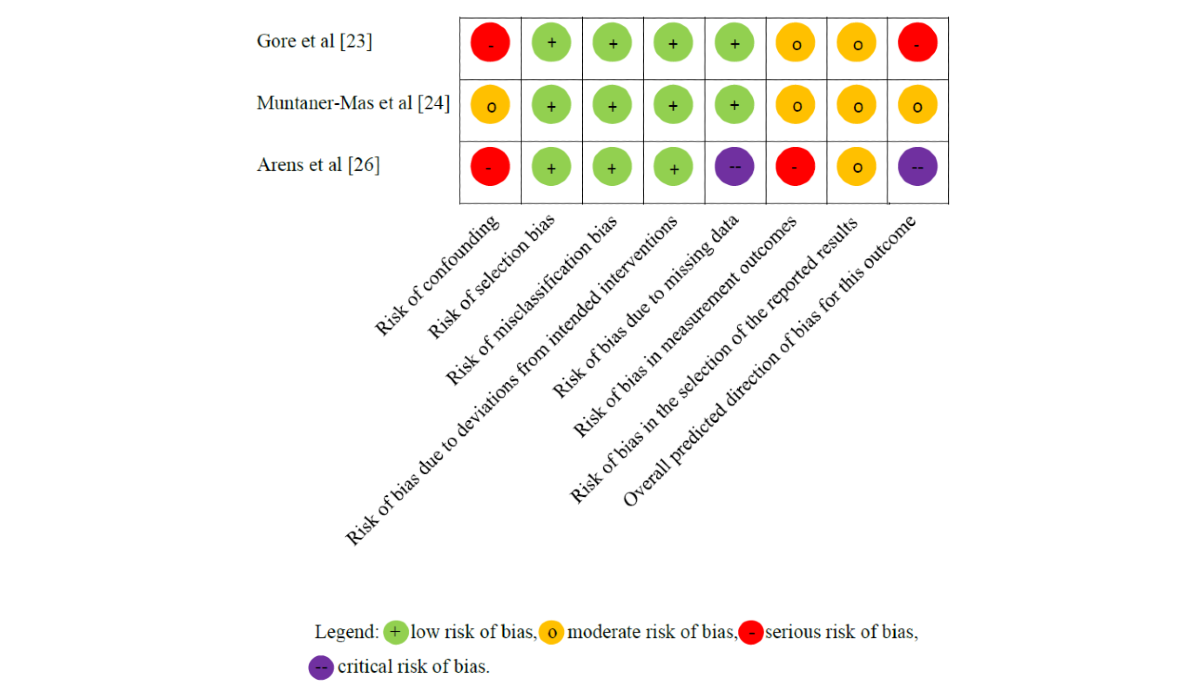
Risk-of-bias summary table for the nonrandomized controlled trials. The upper 2 are cardiovascular disease studies and the lower 1 is a type 2 diabetes study.

Of the 3 non-RCTs [23,24,26], the study by Muntaner-Mas et al [24] was at moderate risk of bias, the study by Gore et al [23] was at high risk of bias, and the study by Arens et al [26] was at critical risk of bias. Figure 4 summarizes the risk of bias for the non-RCTs. The biggest issue with the study by Arens et al [26] was that missing data were not handled adequately, putting the study at critical risk of bias. We assessed the study by Gore et al [23] to be at high risk of confounding because some of the measurements that were used to control for confounding were based on nonvalidated questionnaires.

## Discussion

### Principal Findings

We identified only a small number (n=9) of articles that fulfilled the preset inclusion and exclusion criteria. We assessed most of the studies to be at high risk of bias. Additionally, 3 studies were underpowered (sample size <100), and 2 studies had short follow-up times (<6 months). Ideally, to show the effectiveness in reducing the risk of CVD or T2DM, the studies should have reported disease incidence rates. The only study that did this was that by Ramachandran et al [31], with their primary outcome being a decrease of T2DM incidence due to the SMS text messaging intervention over 2 years. Block et al [28] reported the percentage of people with metabolic syndrome as defined by the International Diabetes Federation Task Force on Epidemiology and Prevention [32]. Block et al [28] also measured change in the Framingham 8-year diabetes risk score [33], and Gore et al [23] measured change in the Framingham 10-year CVD risk score [20]. All other studies reported single risk factors rather than multivariable absolute risk of disease. None of the identified studies directly targeted tobacco smoking cessation or responsible alcohol intake. Rubinstein et al [25] mentioned in their article that their original protocol included both lifestyle factors, but they were later excluded. According to the authors, alcohol intake was considered a sensitive matter requiring face-to-face interactions, while tobacco smoking was excluded because supposedly, compared with physical activity and diet, it had less effect on the onset of hypertension and was more difficult to target via a mobile health intervention [25]. Overall, there were some positive findings suggesting that mobile health-based interventions can achieve at least small to moderate reductions in CVD and T2DM risk, although these were based on weak evidence.

### Strengths and Limitations

The strength of this literature review was that it followed the PRISMA statement. We systematically searched several databases to identify all relevant published articles. Further, we conducted a manual search through the snowballing technique. For the title and abstract screening, a 10% random sample of all retrieved articles was validated by a second researcher, and 2 reviewers independently performed the full article selection. However, only 1 researcher conducted the database search, the data extraction, and the risk-of-bias assessment. Although we a priori restricted the search to English- and German-language articles, we did not exclude any articles because they were not available in these 2 languages. We did not perform a meta-analysis due to the small number of publications that met the inclusion criteria and the differences in their interventions and outcome measures.

### Comparison With Prior Work

Previous mobile health research has focused more on self-management of chronic diseases than on prevention. In their systematic review and meta-analysis, Wu et al [34] investigated the effectiveness of mobile phone apps for diabetes self-management (including prediabetes, gestational diabetes, type 1 diabetes, and T2DM). They identified 3 studies that targeted prediabetes, 2 of which we also included in this review. The overall conclusion of Wu et al [34] was that there was evidence for the effectiveness of app interventions in T2DM self-management, but not for prediabetes. Lunde et al [35] conducted a systematic review looking at various types of noncommunicable diseases and lifestyle advice. Most of the identified studies (8 out of 9) targeted T2DM patients for whom the authors measured improvements in lifestyle factors, particularly reduced glycated hemoglobin levels (in 5 of the 8 studies). For CVD patients, Lunde et al [35] found only 2 relevant articles, and these were without statistically significant improvements in any of the outcomes of interest (weight, BMI, waist circumference, physical activity, and quality of life). A systematic review by Coorey et al [36] focused on self-management of CVD via mobile apps, in which they concluded that short-term improvements in behavior and risk factors were possible but there was insufficient evidence for long-term effects. Alessa et al [37] reported from their systematic review of 21 studies that mobile apps could reduce blood pressure, although the evidence originated mainly from studies that had a high risk of bias.

Palmer et al [14] conducted a systematic review of noncommunicable disease prevention through smoking cessation, alcohol reduction, physical activity, and diet using mobile technology. In total, they found 71 articles, but only 2 of the studies were aimed at the combination of physical activity, diet, and smoking cessation, with both studies targeting secondary prevention of CVD. Among the studies they reviewed, 8 RCTs focused on alcohol reduction but did not include any other lifestyle advice, with these studies specifically targeting heavy drinkers [14]. In general, it appears that many interventions are designed to provide advice for 1 or 2 behavioral risk factors that are associated with increased chronic disease risk, whereas there were only a few evaluation studies of comprehensive mobile health interventions addressing the 4 common behavioral risk factors (ie, tobacco smoking, excessive alcohol consumption, physical inactivity, and poor diet) [14]. Noble et al [13] stated in their systematic review that there were clustering patterns between the 4 behavioral risk factors—tobacco smoking, excessive alcohol consumption, physical inactivity, and poor diet—which indicated similar or the same reasons causing these behaviors. Hence, the authors suggested that future interventions should apply a holistic approach instead of targeting single risk factors. Similarly, Geller et al [38] called for future research studies to focus on improved lifestyles, meaning a change in multiple health behaviors rather than 1, even if it might be harder to achieve. Meader et al [39] reported in their systematic review that targeting smoking simultaneously with other behaviors resulted in negative outcomes for diet and physical activity, suggesting that it might be more beneficial to apply a sequential approach. In a Cochrane review published in 2016, Whittaker et al [40] stated that studies have demonstrated that mobile phone-based interventions can be effective in achieving smoking cessation over 6 months, particularly SMS text messaging in high-income countries.

### Implications and Future Directions

Most studies that were conducted according to the review’s inclusion criteria were at high risk of bias. This review only considered studies of multirisk factor interventions, which resulted in only 9 studies being included. There is a lack of research evaluating interventions that address the 4 common behavioral risk factors (ie, tobacco smoking, excessive alcohol consumption, physical inactivity, and poor diet) in a single mobile health intervention. Researchers may have preferred to focus on 1 risk factor at a time due to simplicity for participants and clarity of intervention-outcome relationships. Hence, future studies should further explore the use of mobile technology for primary disease prevention, by applying a rigorous study design.

### Conclusions

According to the findings of this systematic review, evidence for the effectiveness of mobile health-based interventions in reducing the risk of CVD and T2DM is scarce due to the quality of the included studies and the small effects that were measured. This highlights the need for further high-quality research to investigate the potential of mobile health interventions.

## Appendix

Multimedia Appendix 1 Search strategy.

## Abbreviations

CVD: cardiovascular disease
PRISMA: Preferred Reporting Items for Systematic Reviews and Meta-Analyses
PROSPERO: International Prospective Register of Systematic Reviews
RCT: randomized controlled trial
T2DM: type 2 diabetes mellitus
WHO: World Health Organization
