# Supplementary material for: Primary Prevention of Cardiovascular Disease and Type 2 Diabetes Mellitus Using Mobile Health Technology: Systematic Review of the Literature
Source: J Med Internet Res. 2020 Oct 29;22(10):e21159. doi: 10.2196/21159 (PMC7661239; doi:10.2196/21159)
Supplement: Multimedia Appendix 1 [file jmir_v22i10e21159_app1.doc]

*Search strategies for different databases.*

| Database | Search strategy for CVD^a^ | Search strategy for T2DM^b^ |
| --- | --- | --- |
| **Embase** | ((CVD or "cardiovascular disease") and ("smartphone app*" or mhealth or "mobile health" or "mobile app*" or "smartphone utili*") and (prevent* or risk reduc* or lifestyle or change or intervention or behavio*)).af. | ("type 2 diabetes" and ("smartphone app*" or mhealth or "mobile health" or "mobile app*" or "smartphone utili*") and (prevent* or risk reduc* or lifestyle or change or intervention or behavio*)).af. |
| **Scopus** | TITLE-ABS-KEY ((CVD OR cardiovascular) AND ("smartphone app*" OR mhealth OR "mobile health" OR "mobile app*" OR "smartphone utili*") AND (prevent* OR risk AND reduc* OR lifestyle OR change OR intervention OR behavio*)) | TITLE-ABS-KEY (("type 2 diabetes") AND ("smartphone app*" OR mhealth OR "mobile health" OR "mobile app*" OR "smartphone utili*") AND (prevent* OR risk AND reduc* OR lifestyle OR change OR intervention OR behavio*)) |
| **Science Direct** | Title, abstract, keywords: (CVD OR "cardiovascular disease") AND ("smartphone app" OR mhealth OR "mobile health" OR "mobile app") | Title, abstract, keywords: (CVD OR "cardiovascular disease") AND ("smartphone app" OR mhealth OR "mobile health" OR "mobile app") |
| **CINAHL** | (CVD OR "cardiovascular disease") AND ("smartphone app*" OR mhealth OR "mobile health" OR "mobile app*" OR "smartphone utili*") AND (prevent* OR risk AND reduc* OR lifestyle OR change OR intervention OR behavio*) | type 2 diabetes AND ("smartphone app*" OR mhealth OR "mobile health" OR "mobile app*" OR "smartphone utili*") AND (prevent* OR risk AND reduc* OR lifestyle OR change OR intervention OR behavio*) |
| **Medline** | ((CVD or "cardiovascular disease") and ("smartphone app*" or mhealth or "mobile health" or "mobile app*" or "smartphone utili*") and (prevent* or risk reduc* or lifestyle or change or intervention or behavio*)).af. | ("type 2 diabetes" and ("smartphone app*" or mhealth or "mobile health" or "mobile app*" or "smartphone utili*") and (prevent* or risk reduc* or lifestyle or change or intervention or behavio*)).af. |
| **Proquest**  **(Search limited to scholarly journals)** | noft(CVD or "cardiovascular disease") AND ( noft("smartphone app*") OR noft(mhealth) OR noft("mobile health") OR noft("mobile app*") OR noft("smartphone utili*")) AND (noft(prevent*) OR noft(risk) AND noft(reduc*) OR noft(lifestyle) OR noft(change) OR noft(intervention) OR noft(behavio*)) | noft(CVD or "cardiovascular disease") AND (noft("smartphone app*") OR noft(mhealth) OR noft("mobile health") OR noft("mobile app*") OR noft("smartphone utili*")) AND (noft(prevent*) OR noft(risk) AND noft(reduc*) OR noft(lifestyle) OR noft(change) OR noft(intervention) OR noft(behavio*)) |
| **Engineering Village** | (CVD OR cardiovascular) AND ("smartphone app*" OR mhealth OR "mobile health" OR "mobile app*" OR "smartphone utili*") AND (prevent* OR risk AND reduc* OR lifestyle OR change OR intervention OR behavio*) | (CVD OR cardiovascular) AND ("smartphone app*" OR mhealth OR "mobile health" OR "mobile app*" OR "smartphone utili*") AND (prevent* OR risk AND reduc* OR lifestyle OR change OR intervention OR behavio*) |

^a^CVD: cardiovascular disease.

^b^T2DM: type 2 diabetes mellitus.
